# Chromosome 12q24.31-q24.33 deletion causes multiple dysmorphic features and developmental delay: First mosaic patient and overview of the phenotype related to 12q24qter defects

**DOI:** 10.1186/1755-8166-4-9

**Published:** 2011-04-02

**Authors:** Jawaher Al-Zahrani, Naji Al-Dosari, Nada AbuDheim, Tarfa A Alshidi, Dilek Colak, Ola Al-Habit, Ali Al-Odaib, Nadia Sakati, Brian Meyer, Pinar T Ozand, Namik Kaya

**Affiliations:** 1 Department of Genetics, King Faisal Specialist Hospital and Research Centre, Riyadh, 11211, Saudi Arabia; 2 Department of Zoology, School of Basic Sciences, King Saud University, Riyadh, 11211, Saudi Arabia; 3 Department of Biostatistics, Epidemiology and Scientific Computing, King Faisal Specialist Hospital and Research Centre, Riyadh, 11211, Saudi Arabia; 4 Department of Pediatrics, King Faisal Specialist Hospital and Research Centre, Riyadh, 11211, Saudi Arabia; 5 Yildiz Technical University, Besiktas, 34349, Istanbul, Turkey

## Abstract

**Background:**

Genomic imbalances of the 12q telomere are rare; only a few patients having 12q24.31-q24.33 deletions were reported. Interestingly none of these were mosaic. Although some attempts have been made to establish phenotype/genotype interaction for the deletions in this region, no clear relationship has been established to date.

**Results:**

We have clinically screened more than 100 patients with dysmorphic features, mental retardation and normal karyotype using high density oligo array-CGH (aCGH) and identified a ~9.2 Mb hemizygous interstitial deletion at the 12q telomere (Chromosome 12: 46,XY,del(12)(q24.31q24.33) in a severely developmentally retarded patient having dysmorphic features such as low set ears, microcephaly, undescended testicles, bent elbow, kyphoscoliosis, and micropenis. Parents were found to be not carriers. MLPA experiments confirmed the aCGH result. Interphase FISH revealed mosaicism in cultured peripheral blood lymphocytes.

**Conclusions:**

Since conventional G-Banding technique missed the abnormality; this work re-confirms that any child with unexplained developmental delay and systemic involvement should be studied by aCGH techniques. The FISH technique, however, would still be useful to further delineate the research work and identify such rare mosaicism. Among the 52 deleted genes, *P2RX2, ULK1, FZD10, RAN, NCOR2 STX2, TESC, FBXW8*, and *TBX3 *are noteworthy since they may have a role in observed phenotype.

## Background

Genomic imbalances certainly are major causes of congenital and developmental abnormalities. These include dysmorphia, mental retardation, developmental delay, and multiple congenital anomalies. Some of these genetic anomalies causing such phenotypes can be various and some of these are associated to telomeric/subtelomeric deletions. Among these chromosome 12q24.31-q24.33 telomeric/subtelomeric deletions are rare and only a few patients have been reported previously [1-3]; interestingly none of these were mosaic. Some attempts were made to establish phenotype-genotype correlation [1], no clear relationship could be found. Genes such as *RAN, P2RX2, FZD10*, and *ULK1 *were mentioned as likely candidate genes implicated in the clinical features of the patients reported [1]. In this report a detailed clinical description and molecular cytogenetics analysis of a patient with de novo 12q interstitial deletion is presented. The deleted region contains 52 annotated genes. Among these *P2RX2, ULK1, FZD10, RAN, NCOR2, STX2, TESC, FBXW8*, and *TBX3 *are noteworthy. The function of these genes and some others in 12q24.31-q24.33 region as well as patients having 12q24 related abnormalities were overviewed and discussed to better understand the clinical phenotype.

## Results

### Clinical Details of the Patient

**Figure 1:**
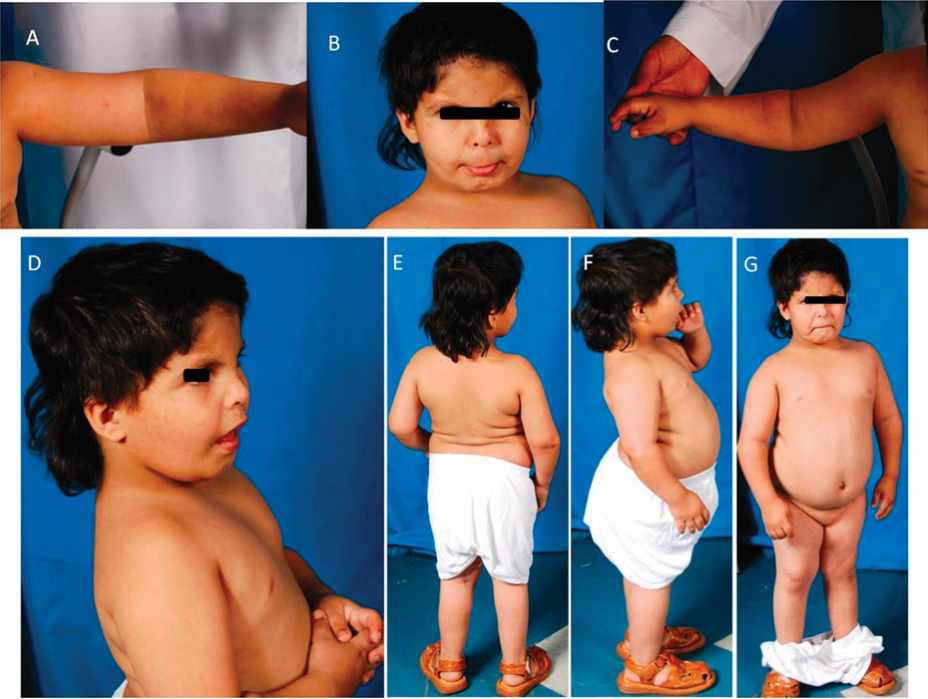
**Patient photos**. Photos showing the patient and clinical phenotypes: A) Left arm showing bent elbow, B) Face indicating long philtrum of upper lip, C) Right arm with bent elbow, D) Facial features including small ear lobe from left side, E-F) Full body indicating scoliosis, short stature, micro-retrognathia, respectively.

**Figure 2:**
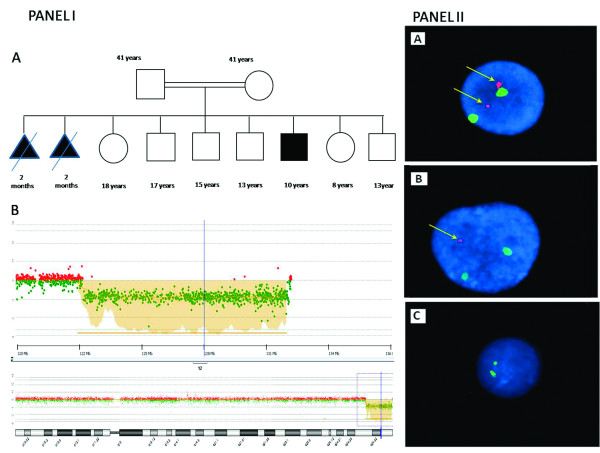
**Family pedigree, aCGH data and FISH results**. Panel I is presenting the family pedigree and aCGH data analysis results. **A**. The pedigree indicates presence of two miscarriages, six healthy family members (two girls and four boys) and one affected boy **B**. aCGH data showing the largest deletion found on chromosome 12q telomere. The deletion is nearly 9.2 Mb in size and starts from bases of 123097890 and extends to the near end of the chromosome 12 and therefore, is considered as a novel telomeric deletion. Similar deletions limited to a few patients were previously reported but the breakpoints and sizes are different. Panel II is depicting three different cells based on interphase FISH results. **A**. Interphase FISH showing the wild type cell with four signals (two telomeric red and two centromeric green signals). **B**. A cell with hemizygosity indicated by three signals based on the FISH experiments. **C**. The FISH result depicting only two green signals in a cell indicating nullisomy for chromosome 12q24.31-q24.33.

The patient (Figure 1A-G), a 10-year-old boy, was born in 2000. Pregnancy and delivery were normal with no history of antenatal and perinatal complications. Family history indicated the parents to be first cousins. They had four normal sons and two normal daughters. There were two miscarriages (Figure 2 Panel I A). No chronic diseases or congenital anomalies exist in the family. The patient was hospitalized twice with chest infection during early infancy. Early global delay of developmental milestones was present. He sat at 10 months and walked at two years of age. He spoke only two words.

At 5 9/12 years of age his height, weight and head circumference were 83 cm, 13 kg and 47.5 cm. These represented the medians of 19/69, 24/69 and 14/69 respectively; and all were well below 3^rd ^percentile. He had micrognathia, small ears, impalpable testes, elbow deformity and kyphoscoliosis. He had a short and small penis in addition to the undescended testicles. The system examination including chest, heart, abdomen and central and peripheral nervous system were all normal. The blood count, renal and hepatic profile and other chemistries were within normal limits. The ECG was normal. A karyotyping and FISH examination for 22q11.2 region were normal. The diagnosis of panhypopituitarism was entertained. The IGF1 was 24 mEq/Lv. The TS and TSH were normal. Up to seven years of age, he showed poor speech, developmental delay and growth retardation; his height was 87 cm, at 2 years chronological age was 6 years. At present he is 10-year-old with no improvement in his clinical findings (Figure 1A-G).

### Molecular Findings

Standard G-banding performed on patient's lymphocytes showed no chromosomal abnormality. The Agilent's Human Genome CGH Microarray 244A array revealed that telomere of 12q showed an interstitial hemizygous deletion starting from the middle of 12q24.31 Mb (132293878 bp) to the near end of the q arm (123065364 bp) (Chromosome 12: 46,XY,del(12)(q24.31q24.33) on the patient's DNA (Figure 2 Panel I B). No other chromosomal imbalances were identified. Parents were also tested by the same array type but yielded no such deletion. The aCGH result was confirmed by MLPA assay (data not shown). A detailed breakpoint analysis is given in Additional file 1. Two independent interphase FISH experiments were performed on cultured peripheral blood lymphocytes from the patient using commercially available 12q telomeric and chr12 centromeric FISH probes from Vysis Inc. Each time 250 cells were counted at interphase. These results indicated mosacism with three different cell types a) wild type cells having four signals two centromeric and two telomeric (44.5%), b) mutant cells with hemizygous 12q telomeric deletion (30%), c) mutant cells with homozygous 12q telomeric deletion in the patient (25.5%) (Figure 2 Panel II A, B, C, respectively). As a next step we wanted to test other tissue types to see if the mosaicism is restricted to the lymphocytes. Unfortunately we were not able to reach to the family to collect more samples to further analyze the mosaicism.

## Discussion

Chromosome abnormalities involving the telomere of chromosome 12 are rare. A comprehensive subtelomere FISH analysis was conducted among 11,688 patients with developmental disabilities [4]. 357 instances of the abnormalities were detected such as duplications, deletions, and translocations; out of that 357 only three patients had chromosome 12q telomere abnormality.

In another study of micro-array based molecular karyotyping as preimplantation genetic screening (PGS) on 24 h blastomers indicated that 46.2% of 134 blastomers were mosaic euploid. Meiotic trisomies were negatively predictive of mosaic euploid embryos [5]. The reason for this high frequency of mosaicism at cleavage stage of development is unknown. One theory is that mosaicism originates from chromosomally abnormal oocytes as a result of trisomy rescue [6]. Chromosome mosaicism could also be seen in 1-2% of chorionic villus biopsies [7]. In their investigation of 15,109 CVS a mosaic condition was found in 203 instances. This was speculated to be either a mitotic originated event owing to a postzygotic non-dysjunction resulting a trisomic cell line in an originally normal conceptus or a meiotic originated event due to postzygotic loss of one chromosome an initially trisomic conceptus namely atrisomy rescue. However, this placental mosaicism extended only in 12.8% of instances from placenta into fetal tissues. A study in mouse indicated that aneuploid embryos could implant in intrauterine tissue and initiate gastrulation but quickly degrade and die [8]. Embryo viability due to chromosomal mosaicism is mediated by p53-independent apoptotic mechanism. These observations suggest that chromosome mosaicism is not uncommon in pregnancies; although the etiology is unknown it might be a "rescue mechanism" for a trisomic cell line. Among mosaic cell lines, abnormality of 12q terminus should also be rare since in chromosome 12q there is a local telomere-induced suppression of recombination.

There have been only four instances of deletions involving 12q24.31-q24.33 region in the literature. The first instance of such deletions was a female with mild non-familial mental retardation (MR), but no further clinical features were reported [3]. The deletion was not visible by retrospective high-resolution G-banding analysis. Using FISH, the size of the deletion was estimated to be between 3 and 6 Mb from the telomere. The second two instances of 12q subtelomeric deletions were reported with full clinical description and fine mapping [1]. Additionally two boys, a 9 months-old, with abnormal genitalia [3] and the other whose interstitial deletion did not include 12q24.33, without abnormal genitalia were reported [2]. The features of these patients are listed in Additional file 2 and briefly include mild MR, developmental delay, some facial, hand and foot abnormalities, obesity, behavioral manifestations, as food seeking, high tolerance for pain, and various genital abnormalities [1-3].

The approximate deletion size was 9,228,514bp (Additional file 1). This deletion is larger than those that contained 14 and 22 known genes reported before [1] and comprises at least 52 annotated genes according to the NCBI Map Viewer (Build 36.2). The 12q24-qter region as a block contains more than 300 genes. Among them 49 are hypothetical genes and another 43 genes are thought to be relevant to the phenotype (Additional file 3).

In the literature only one report detailed the genes involved [1]. Their aCGH analysis suggested a possible role of *P2RX2 *(purinergic receptor P2X2 isoform 1) and *ULK1 *(UNC51-like kinase) genes in patient 1 and RAS related protein (RAN) and Frizzled 10 (*FZD10*) in the second patient. The main consequences of this deletion were in the nervous system in both patients[1]. Since the gene products of both *P2RX2 *and *ULK1 *have been demonstrated to be involved in neuronal function, their haploinsufficiency might be implicated in MR and behavioral problems that were also observed in our patient.

Terminal region of chromosome 12 q is involved in the normal development of male genitalia [3] and most patients with such deletion had abnormalities of this organ (Additional file 2). It appears that *STX2, TESC *and *FBXW8 *genes might be responsible for defective testicular development and *TBX3 *for small penis. Another critical gene is *NCOR 2 *in the region deleted in the present patient might be involved in the phenotype. It has many functions; including its repressive function through regulation of chromatin [9]. It was demonstrated NCOR2 is a co-regulator for the androgen receptor (AR) and through an intricate mechanism NCOR2 modulates the androgen receptor activity [10]. It is involved in transcriptional regulation by both agonist- and antagonist-bound AR and that it regulates the magnitude of hormone response [11]. NCOR2 involves in progression of neural stem cells into neurons by mediating repression of H3K27 demethylase through JMJD3 and plays a critical role in forebrain development and neural stem cell maintenance. It is shown in the NCOR2 gene-knockout mice that both retinoic-acid-dependent and Notch-dependent forebrain development require NCOR2 [12]. These might explain gonadal and CNS findings in our patient.

In the 12q24 area there are other genes that might be involved in the phenotype (Additional file 3) including *THRAP2, TBX5 *and *PTPN11 *for cardiac and great vessel anomalies; *TBX5, CMKLR1*, and *TRPV4 *for skeletal defects; *PRKAB1, GPR109A*, and *GPR109B *for increased hunger and obesity. At least seven genes are closely related to the function of nervous system: *TECT1, MSI1, DYNLL1, SRRM4, DNAH10, NOS 1, P2RX4 *besides those mentioned before.

## Conclusions

In conclusion, it is difficult to speculate for a mosaic case as to which genes likely to contribute to a clinical phenotype. This is particularly true for our patient since only one out of three cells was normal, the other two were either hemizygous or homozygous for the deletion. However, generally speaking, based on the function and annotation of the genes from the literature one can speculate that *P2RX2*, *ULK1*, *RAN*, *FZD10*, *NCOR2, STX2, TESC, FBXW8, TBX3*, and other 15 genes are likely to be responsible for the phenotype to a certain extent.

## Methods

### Patients, Sample Collection, Cell Culture and DNA Isolation

After clinical evaluation including dysmorphology examination, patients and some of their relatives were recruited under the KFSHRC IRB-approved protocol (RAC# 2040042 and 2060035) and consented for their participation to the study. The blood samples were collected into different tubes for DNA, RNA isolation and cell culture. Nucleic acids isolations and cell culture were according to standard protocols [13].

### Array CGH Experiment

Human Genome CGH Microarray Kit 244A (Agilent Technologies, Santa Clara, CA, USA) was used in the aCGH experiments. Genomic DNA preparation, labeling, hybridization, scanning, image extraction, data generation, and visualization were all performed according to the manufacturer's instructions (Agilent Inc.).

### Cytogenetics Analysis and Interphase FISH Experiment

Chromosome analysis performed on cultured peripheral blood lymphocytes, using standard G banding techniques. Interphase FISH analysis was performed using two commercially available probes from Vysis (A centromeric probe for Chromosome 12, Vysis CEP12-D12Z3), and a telomeric probe, Vysis Telysion 12-VIJyRM2196-LOCUS) on cultured cells according to standard procedures.

### MLPA Assay

SALSA MLPA kit P286 Telomere-11 (MRC-Holland, Amsterdam, Holland) was used for MLPA experiments. Experimental procedures and data analysis were performed according to manufacturer's protocols and guidelines.

## Competing interests

The authors declare that they have no competing interests.

## Authors' contributions

NK was PI of a project (named briefly molecular cytogenetics) in which the samples were collected, and the project was approved by KFSHRC. JA was master student of NK working on the selected samples as part of her thesis and performed most of the experiments and drafted the manuscript with the help of NK. NAs, AA, and TA performed cell cultures and helped JA for FISH experiments. JA performed aCGH experiments with help of NK. NK and DC analyzed the data. PTO and NS clinically examined the patients, and collected the samples. BM and OA helped to analyze FISH and help to calculate cells for mosaicism. All authors approved the manuscript.

## Consent

Consent forms for the project and photos were taken from the parents according to our institution's IRB approval.

## Supplementary Material

Additional file 1 **A detailed proximal and distal breakpoint analysis**. Click here for file

Additional file 2 **Clinical summary of cytogenetic abnormalities involving deletion, duplication, or translocation of 12q telomere**. Click here for file

Additional file 3 **Chromosome 12q24-qter genes relevant to phenotype**. Click here for file

## References

[B1] Niyazov DM Nawaz Z Justice AN Toriello HV Martin CL Adam MP Genotype/phenotype correlations in two patients with 12q subtelomere deletions Am J Med Genet A 2007 143A 2700 2705 10.1002/ajmg.a.32005 17937441

[B2] Plotner PL Smith JL Northrup H Deletion 12q: a second patient with 12q24.31q24.32 deletion Am J Med Genet A 2003 118A 350 352 10.1002/ajmg.a.10232 12687666

[B3] Sathya P Tomkins DJ Freeman V Paes B Nowaczyk MJ De novo deletion 12q: report of a patient with 12q24.31q24.33 deletion Am J Med Genet 1999 84 116 119 10.1002/(SICI)1096-8628(19990521)84:2<116::AID-AJMG6>3.0.CO;2-3 10323735

[B4] Ravnan JB Tepperberg JH Papenhausen P Lamb AN Hedrick J Eash D Ledbetter DH Martin CL Subtelomere FISH analysis of 11 688 cases: an evaluation of the frequency and pattern of subtelomere rearrangements in individuals with developmental disabilities J Med Genet 2006 43 478 489 10.1136/jmg.2005.036350 16199540 PMC2564531

[B5] Johnson DS Gemelos G Baner J Ryan A Cinnioglu C Banjevic M Ross R Alper M Barrett B Frederick J Preclinical validation of a microarray method for full molecular karyotyping of blastomeres in a 24-h protocol Hum Reprod 25 1066 1075 10.1093/humrep/dep452 20100701 PMC2839907

[B6] Kuliev A Verlinsky Y Meiotic and mitotic nondisjunction: lessons from preimplantation genetic diagnosis Hum Reprod Update 2004 10 401 407 10.1093/humupd/dmh036 15319376

[B7] Grati FR Grimi B Frascoli G Di Meco AM Liuti R Milani S Trotta A Dulcetti F Grosso E Miozzo M Confirmation of mosaicism and uniparental disomy in amniocytes, after detection of mosaic chromosome abnormalities in chorionic villi Eur J Hum Genet 2006 14 282 288 10.1038/sj.ejhg.5201564 16418738

[B8] Lightfoot DA Kouznetsova A Mahdy E Wilbertz J Hoog C The fate of mosaic aneuploid embryos during mouse development Dev Biol 2006 289 384 394 10.1016/j.ydbio.2005.11.001 16337934

[B9] Perissi V Jepsen K Glass CK Rosenfeld MG Deconstructing repression: evolving models of co-repressor action Nat Rev Genet 11 109 123 10.1038/nrg2736 20084085

[B10] Liao G Chen LY Zhang A Godavarthy A Xia F Ghosh JC Li H Chen JD Regulation of androgen receptor activity by the nuclear receptor corepressor SMRT J Biol Chem 2003 278 5052 5061 10.1074/jbc.M206374200 12441355

[B11] Yoon HG Wong J The corepressors silencing mediator of retinoid and thyroid hormone receptor and nuclear receptor corepressor are involved in agonist- and antagonist-regulated transcription by androgen receptor Mol Endocrinol 2006 20 1048 1060 10.1210/me.2005-0324 16373395

[B12] Jepsen K Solum D Zhou T McEvilly RJ Kim HJ Glass CK Hermanson O Rosenfeld MG SMRT-mediated repression of an H3K27 demethylase in progression from neural stem cell to neuron Nature 2007 450 415 419 10.1038/nature06270 17928865

[B13] Ozand PT Gascon G al Aqeel A Roberts G Dhalla M Subramanyam SB Prevalence of different types of lysosomal storage diseases in Saudi Arabia J Inherit Metab Dis 1990 13 849 861 10.1007/BF01800209 2079833

