# Supplementary material for: Chromosome 12q24.31-q24.33 deletion causes multiple dysmorphic features and developmental delay: First mosaic patient and overview of the phenotype related to 12q24qter defects
Source: Mol Cytogenet. 2011 Apr 2;4:9. doi: 10.1186/1755-8166-4-9 (PMC3083380; doi:10.1186/1755-8166-4-9)
Supplement: Additional file 2 — Clinical summary of cytogenetic abnormalities involving deletion, duplication, or translocation of 12q telomere. [file 1755-8166-4-9-S2.DOC]

**Additional file 2:** Clinical Summary of cytogenetic abnormalities involving 12q telomere deletions.

| **CYTOGENETIC ABNORMALITY** | **PHENOTYPE** | **REFERENCES** |
| --- | --- | --- |
| **46XY; 12q subtelomeric deletion; 1.6Mb; 14 genes** | 8 years old, borderline MR, delayed language development, food seeking behavior, attention deficit-hyperactivity, high pain threshold, disorder, no facial dysmorphia, additional anterior second hair-whorl, brachydactyly, clinodactyly, obesity. | [1] |
| **46XY; 12q subtelomeric deletion; 4.5 Mb; 22 genes** | 12 years old, left cryptorchidism, multi-cystic left kidney, ectopic right kidney, epicanthal folds, small ears, delayed milestones, moderately intellectually disabled, food seeking behavior, self inflicting behavior. | [1] |
| **46XY; del(12)(q24.31-24.33)** | 9 months old, tracheomalacia, ambiguous genitalia; Dandy-Walker syndrome, coarse face, large anterior fontanella, short palpebral fissure, ptosis of the left eye, thick gums, large tongue, mild generalized hypotonia, bifid scrotum, bilaterally palpable testes, stretched penile length 2.9 cm. (normal > 2.8 cm); long fingers with patulous finger tips; delayed at 9 months could not sit unsupported, nor crawled, nor babbled or cooed. | [2] |
| **46 XY; del(12)(q24.31q24.32)** | 20 month old, developmentally developmental level 10-12 months at/20 months of age, no genital abnormalities, microcephalic, large pubic fat pad, sacral pit, tapering fingers, clinodactyly, pes planovalgus, perimembranous VSD, mild tricuspid regurgitation, secundum ASD, mild biventricular and left atrial dilatation, mild tricuspid regurgitation, facial dysmorphia with large bulbous nose, smooth filtrum, large ears. | [3] |
| **46XY; del12q 24.31-q24.33; mosaic** | 8 years old, severe growth retardation with very low IGF1 level, developmental retardation, dysmorphic face, low-set ears, cryptorchidism with short penis, elbow deformity. | Present patient |

**REFERENCES**

1. Niyazov DM, Nawaz Z, Justice AN, Toriello HV, Martin CL, Adam

2. Sathya P, Tomkins DJ, Freeman V, Paes B, Nowaczyk MJ: De novo deletion 12q: report of a patient with 12q24.31q24.33 deletion. *Am J Med Genet* 1999, 84:116-119.

3. Plotner PL, Smith JL, Northrup H: Deletion 12q: a second patient with 12q24.31q24.32 deletion. *Am J Med Genet A* 2003, 118A:350-352.

MP: Genotype/phenotype correlations in two patients with 12q subtelomere deletions. *Am J Med Genet A* 2007, 143A:2700-2705.
