# Supplementary material for: Chromosome 12q24.31-q24.33 deletion causes multiple dysmorphic features and developmental delay: First mosaic patient and overview of the phenotype related to 12q24qter defects
Source: Mol Cytogenet. 2011 Apr 2;4:9. doi: 10.1186/1755-8166-4-9 (PMC3083380; doi:10.1186/1755-8166-4-9)
Supplement: Additional file 3 — Chromosome 12q24-qter genes relevant to phenotype. [file 1755-8166-4-9-S3.DOC]

**Additional file 3:** Chromosome 12q24-qter genes relevant to phenotype

| **SYMBOL** | **NAME OF THE GENE** | **LOCATION** | **OMIM** | **FUNCTION and PHENOTYPES** |
| --- | --- | --- | --- | --- |
| **ABCB9** | ATP binding casette | 12q24 | **605453** | ATP binding cassette associated with lysosomes |
| **NCOR2** | Nuclear co-receptor suppressor 2 | 12q24 | **600848** | Regulates androgen receptor, development and morphogenesis |
| **P2RX7** | Purinergic receptor 2, ligand gated ion channel 7 | 12q24 | **602566** | Neuronal function |
| **SLC24A6** | Solute carrier family 24 (sodium, potassium calcium exchanger ) | 12q24 | **609841** | Maintains Ca2+ homeostasis by K+ depend3ent Na+ Ca2+ exchange |
| **THRAP2** | Thyroid hormone receptor associated protein 2 | 12q24 | **608771** | Embryonic development, transposition of great vessels, |
| **APPL2** | Adaptor protein | 12q24.1 | **606231** | Link to small GTPase RAB5, remodels nucleosome |
| **CMKLR1** | Chemokine-likereceptor 1 | 12q24.1 | **602351** | Possible role in the inflammatory pathways |
| **PRKAB1** | AMP activated protein kinase | 12q24.1 | **602740** | Likely role in monitoring cellular energy status |
| **PTPN11** | Protein tyrosin phosphatase nonreceptor protein11 | 12q24.1 | **176876** | Plays a regulatory role in various cell signaling events |
| **RPH3A** | Rabphillin 3 a | 12q24.1 | **612159** | Target of RAB3a, exocytosis of neurotransmitters |
| **TBX3** | T-Box 3 | 12q24.1 | **601621** | Plays a role in the anterior/posterior axis of the tetrapod forelimb |
| **TBX5** | T-Box 5 | 12q24.1 | **601620** | Likely role in heart development and specification of limb identity |
| **TECT1** | Tectonic 1 | 12q24.1 | **609863** | Modulates hedge-hog signaling, |
| **TPCN1** | Two-pore segment channel | 12q24.1 | **609666** | Nicotinic acid-ADP receptor signals release of Ca2+ |
| **TRPV4** | Transient receptor potential cation channel Subfamily V, member 4 | 12q24.1 | **605427** | Likely to involve in behavioral responses to osmotic and mechanical stimuli in mammals |
| **STK21** | Serine/protein kinase 21 | 12q24.1q24.3 | **605629** | CNS malformation, defective neurogenesis |
| **MSI1** | Musashi 1 | 12q24.1q24.1 | **603328** | Regulates neural development, decides fate of neurogenitor cells |
| **DYNLL1** | Dynein light chain 1, C3 type 1 | 12q24.2 | **601562** | Inhibits NO Synthase in brain |
| **KSR2** | Kinase suppressor of RAS 2 | 12q24.2 | **610737** | Ras signaling pathways for serine/threonine kinase |
| **TESC** | Tescalcin | 12q24.2 | **611585** | Encodes a protein involved in testicular development |
| **FBXW8** | F-Box and WD40 domaine protein 8 | 12q24.23 | **609073** | F-Box protein, induces phospho-dependent ubiquination of SCF complex |
| **PEPB1** | Phosphodylethanolamin binding protein | 12q24.23 | **604591** | RAF-kinase inhibitor associates with centrosome, regulates the spindle check-point |
| **SRRM4** | Serine/arginine repetitive matrix protein 4 | 12q24.23 | **613103** | Normal neuron development, and neurite extension, located in introns includes minigenes to exons |
| **NOS1** | Neuronal nitric oxide synthase | 12q24.2-q24.31 | **163731** | A neurotransmitter in brain and peripheral CNS |
| **RAN** | Ras-related nuclear protein | 12q24.3 | **601179** | Transfer of RNA and proteins through nuclear pores |
| **UBC** | Ubiquitin C | 12q24.3 | **191340** | Associates with PPAR signaling pathway |
| **ULK1** | Uncr51like kinase 1 | 12q24.3 | **603168** | Neuronal differentiation, axon elongation |
| **BAZ2A** | Bromodomain adjacent to zinc finger domain 2A | 12q24.3-qter | **605682** | Involved in regulating the function of chromatin modeling function of NORC |
| **DNAH10** | Dynein axonemal heavy chain 10 | 12q24.3.1 | **605884** | Axonemal dynein activity; ATPase ATP binding motor of microtubule assembly |
| **GPR81** | G-protein coupled receptor 81 | 12q24.3.1 | **606923** | G-coupled receptor, signal transduction |
| **GPR109A** | G-protein coupled receptor 109A | 12q24.3.1 | **609163** | Nicotinic acid receptor suppresses adipose lipolysis |
| **GPR109B** | G-protein coupled receptor 109B | 12q24.3.1 | **606039** | Adipose tissue nicotinic acid receptor, adipose tissue |
| **SPPL3** | Signal peptide peptidase like 3 | 12q24.3.1 | **608240** | Aspartyl protease cleaves signal peptides |
| **VPS33A** | Vacuolar protein sorting 33a | 12q24.3.1 | **610034** | Mitochondria based apoptotic signal |
| **VPS37B** | Vacuolar protein sorting 37B | 12q24.3.1 | **610037** | Part of human endosomal complex |
| **LHX5** | LIM homeo box protein 5 | 12q24.31-q24.32 | **605992** | Regulates precursor cell proliferation and migration |
| **P2RX4** | Purinergic receptor ion gated channel 4 | 12q24.32 | **600846** | Ionotropic ATP gated ion channel. |
| **ZNF140** | Zinc finger 140 | 1qq24.32-q24.33 | **604082** | Function as transcription repressors |
| **EP400** | P400 SW12/SNF2 related protein | 12q24.33 | **606265** | Chromatin modification |
| **STX2** | Epimorphine (Synraxin2) | 12q24.33 | **132350** | Testicular development and spermatogenesis |
| **MMP17** | Matrix metalloproteinase 17 | 12q24.33 | **602285** | Membrane bound MMP peptidoglycan metabolism |
